# Phytochemical Characterization and Bioactivity of Different Honey Samples Collected in the Pre-Saharan Region in Algeria

**DOI:** 10.3390/life12070927

**Published:** 2022-06-21

**Authors:** Safia Ben Amor, Scherazad Mekious, Leila Allal Benfekih, Magda H. Abdellattif, Walid Boussebaa, Faisal A. Almalki, Taibi Ben Hadda, Sarkar M. A. Kawsar

**Affiliations:** 1 Laboratory for Research on Medicinal and Aromatic Plants, Faculty of Nature Sciences and Life, Saad Dahlab University, Blida 1, Route de Soumâa, Blida 09000, Algeria; mekious.sch.uni.djelfa@gmail.com (S.M.); leilaallalbenfekih@yahoo.fr (L.A.B.); 2 Faculty of Nature Sciences and Life, Ziane Achour University, Djelfa 17000, Algeria; 3 Department of Chemistry, College of Science, Taif University, Al-Haweiah, P.O. Box 11099, Taif 21944, Saudi Arabia; m.hasan@tu.edu.sa; 4 Scientific and Technical Research Center in Physico-Chemical Analysis, Headquarters Ex-Pasna Industrial Zone, Bou-Ismail CP, Tipaza 42004, Algeria; walidboussebaa@gmail.com; 5 Department of Pharmaceutical Chemistry, Faculty of Pharmacy, Umm Al-Qura University, Makkah 21955, Saudi Arabia; malkifaisal2@gmail.com (F.A.A.); taibi.ben.hadda@gmail.com (T.B.H.); 6 Laboratory of Bioresources, Biotechnology, Ethnopharmacology and Health, Faculty of Sciences, Université Mohamed Premier, BV Mohammed VI, BP 717, Oujda 60000, Morocco; 7 Laboratory of Carbohydrate and Nucleoside Chemistry, Department of Chemistry, Faculty of Science, University of Chittagong, Chittagong 4331, Bangladesh

**Keywords:** honey, melissopalynology, physicochemical analysis, LC-MS-MS, antioxidant test, antimicrobial activity

## Abstract

Despite the challenging conditions in the pre-Saharan areas of Algeria, such as weak plant cover and a harsh climate, beekeeping is being developed and spread. In the present work, honey samples collected from ten locations in the El Oued region were examined during the spring of 2021. A melissopalynological analysis was carried out, followed by a floristic investigation. The 10 honey samples were also investigated for their physicochemical properties and antioxidant and antibacterial activity against five strains: *Escherichia coli*, *Staphylococcus aureus*, *Bacillus subtilus*, *Listeria innocua,* and *Micrococcus luteus.* The floristic analysis found 65 species belonging to 33 botanical families, with a dominance of the Asteraceae family accounting for 18.461% of the total. The melissopalynological study revealed only one monofloral honey (*Ziziphus lotus*), whereas the nine others were multi-floral. The honey’s color changed from light to dark amber, and most tested honey was of high quality, fulfilling international criteria. The total phenol and flavonoid contents varied considerably amongst the various honey samples. Furthermore, LC-MS-MS phenolic profile analysis identified the presence of 20 chemicals, of which only three phenols were found in all honey types. Antioxidant capacity analyzed with FRAP test and antiradical activities against DPPH differed from one honey sample to another. Moreover, a significant correlation was recorded between the antioxidant activity, honey’s color, polyphenol, and flavonoid contents. The *S. aureus* strain was the most sensitive regarding honey antibacterial activity, while *M. luteus* and *B. subtilis* strains were only moderately sensitive.

## 1. Introduction

Honey is a natural product with a complex chemical composition, and it is also the only and the most well-known sweetener that can be consumed raw by humans [1]. Bees collect nectar and pollen from plants and produce honey, which has been revered for centuries for its nutritional and therapeutic properties [2]. Honey has been resurrected as a therapy for burns, gastrointestinal diseases, asthma, infected wounds, and skin ulcers in human and animal medicine [3,4].

Honey contains several constituents of small amounts, such as minerals, free amino acids, proteins, vitamins, enzymes, organic acids, flavonoids, phenolic acids, and other organic acids in addition to other phytochemicals compounds [5]. The amount of these components is determined by several factors, including the honey’s geographical origin, floral source, meteorological circumstances, any treatments applied [6], and seasonality [7]. Honey’s composition can be affected by processing, handling, and storage [8]. The quality of honey also depends on floral resources and the treatment of the beekeepers [9].

Honey’s botanical and geographical origins have traditionally been determined by evaluating pollen quality and quantity and organoleptic and physicochemical testing. In addition, data derived from the sensory profile, bioactive components, and novel methods of investigation should be added to this information [10,11].

Water content, sugar reduction, sucrose, insoluble matter, ash, free acid, pH, electrical conductivity, specific rotation, and sensory and microbiological properties are the basis for the quality assessment of honey [12,13]. Honey’s components have a variety of beneficial biological actions, such as antioxidant, antifungal, antibacterial and antiviral, anti-browning effects, and antioxidants effects in natural foodstuffs [14,15]. Various studies have demonstrated that antioxidant activity highly correlates to total phenolic levels [16]. Moreover, darker honey has been reported to have a higher total phenolic content and thus more significant antioxidant activity [17]. Honey’s composition includes various components, including hydrogen peroxide and polyphenols, and is also strongly linked to antibacterial activity [18]. The latter is diverse and yet not fully understood. Several components of honey have been shown to have a critical role in honey’s antibacterial effects. Honey’s ability to fight different sorts of microorganisms is determined by various variables, including the kind and natural structure of the nectar and the environmental circumstances in which the bees were raised [19].

Despite severe environmental conditions and poor plant cover, beekeeping is being developed and promoted in pre-Saharan regions. The western and central parts of Algeria are the focus of studies on the kinds of honey of the Saharan region [20]. However, in our perspective, no work has addressed the research of kinds of honey from the south-eastern region of the country, particularly those bordering Tunisia. In the Algerian Sahara, the inhabitants frequently use Saharan honey as a remedy for many infections because of its medicinal attributes and higher efficacy than in North Africa [21]. This present study aims to investigate honey plants in a pre-Saharan area of Algeria and appraise their honey using physicochemical parameters, pollen and color analyses, phenolic compounds and their dosages, and an evaluation of their antioxidant activities. Honey’s antibacterial activity was also assessed against Gram-positive and Gram-negative strains.

## 2. Materials and Methods

### 2.1. Honey Samples Origin

**Figure 1:**
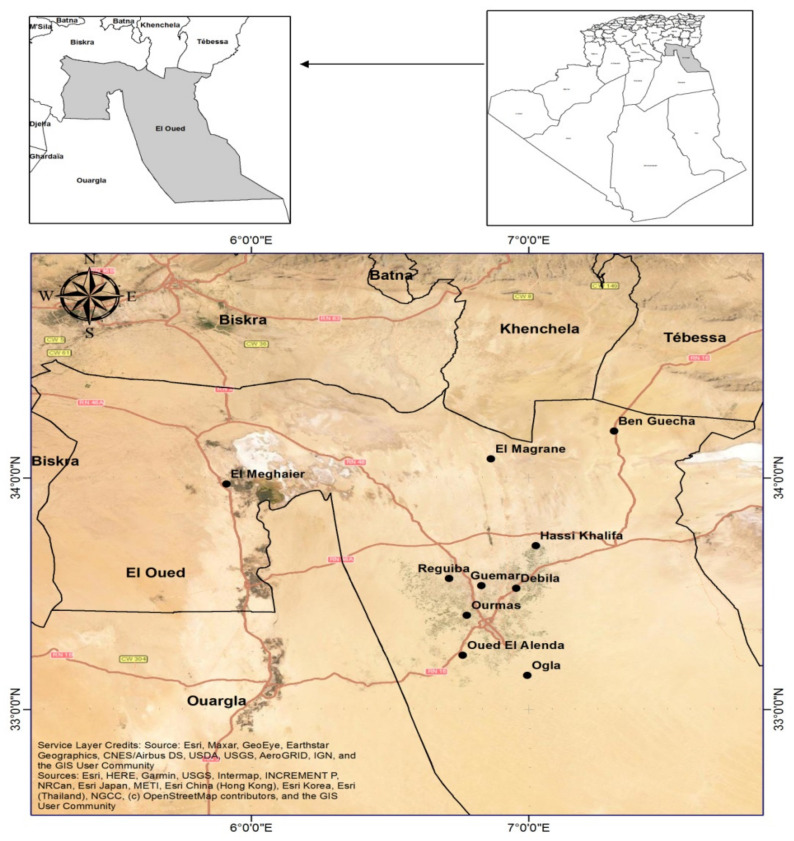
Distribution of the study sites in El Oued region.

The current study was conducted on ten honey samples obtained from *Apis mellifera intermission* apiaries installed in various localities in the El-Oued region, a 77 km^2^ area located in the northeastern Sahara of Algeria (Low Sahara basin) (Figure 1 and Table 1), and characterized by a dry to hyper-arid climate. Ten sites, of which two are natural and eight cultivated, were selected according to their distance from each other and the beekeepers’ activity. Nine sites are located in the Oued Souf region, whereas the tenth site is situated in the Oued Righ area. The samples were collected at the end of May 2021, and all honey samples were kept at 4 °C until the analysis.

### 2.2. Vegetation Study

The flora investigation and plant cover were conducted in the spring using the quadrat technique in a 500 m^2^ area [22] by installing quadrat plots every 200 m in the bee-foraging radius. From November until May, flowering plants were collected weekly and haphazardly at each study site. According to our observations and beekeepers’ information, day-long (from 8 h to 10 h and from 12 h to15 h 30 h) melliferous plants foraged by honey bees were divided into three percentages cover categories: frequent (>60%), average (*˂*60% to >30%), and less often (<30%) [22]. The identification of plant flowering species was determined based on Ozenda [23] and the flora of North Africa [24].

### 2.3. Pollen Analysis

The melissopalynological qualitative study was reported by Louveaux et al. [25]. A sample of 10 g of honey was dissolved in 20 mL of acidic water 5% (5 g H_2_SO_4_ per one liter of distilled water), and the mixture was centrifuged at 3500 rpm for 10 min, after which the supernatant was discarded. The precipitate was soaked in 10 mL of distilled water and centrifuged again for 5 min. The deposit containing the pollen grains was spread on a slide. After drying, a drop of glycerin gelatin was added to it, where it was then covered with a cover slip for identification. To determine frequency classes, 500 pollen grains were counted per sample. The four following categories were used for frequency classes: predominant pollen (>45%) of pollen grains counted, secondary pollen (16–45%), important minor pollen (3–15%), and minor pollen (1–3%) [25,26]. The pollen grains were observed using an optical microscope, and pollen types were identified using a reference collection collected from the beehives’ area. We also used the pollen atlas established by Riccardeli D’Albore [27].

### 2.4. Physicochemical Analyses

The physicochemical characteristics of the honey samples were determined three times using the International Honey Commission methods (IHC) [28]. First, water content was determined by using a refractometric method. All honey samples were placed in firmly closed, sterile vials and were incubated in a water bath at 50 degrees Celsius for 30 min, then cooled at room temperature until 20 °C. Refractive index values were then measured, and a standard CHATAWAY table was used to calculate the associated percent humidity (g/100 g honey). Second, the pH of honey was measured using a pH meter whereby 10 g of honey at a temperature of 20 °C was dissolved in ultra-pure water under a magnetic agitator to avoid the precipitation of sugars and maintain the solution homogeneity. Third, the titrimetric approach was employed to determine free acidity. After dissolving 10 g of honey with 75 mL of pure water, the volume of 0.1 M NaOH was added to the resulting solution until reaching a pH of 8.5. The results were expressed in meq/kg. To determine electrical conductivity, a conductivity bridge was employed to analyze a 20% (w/v) distilled water–honey solution [28]. The electrical conductivity values were given in mS/cm. To determine the color of honey, solutions of 50% were heated to 50 degrees until the sugars were wholly dissolved. Absorbance was measured at 635 nm, and absorbance values were classified according to the Pfund scale [29].

### 2.5. Total Polyphenols and Flavonoids Content

The Folin–Ciocalteu procedure was employed to measure the overall phenol content of the sampled honey with a few modifications [30]. Each gram of each honey sample was diluted into 10 mL of distilled water, filtered through Whatman paper No.1, then mixed with 2.5 mL of 0.2 N Folin–Ciocalteu reagents. The mixture was left to stand for 5 min. Then, 2 mL of 20% aqueous sodium carbonate solution (Na_2_CO_3_) was added. The reaction was kept in the dark at room temperature for 2 h, and the absorbance was measured with a spectrophotometer at 760 nm. Total phenolic content was calculated using the linear regression equation of the plotted calibration curve of gallic acid using the linear regression equation of the plotted calibration curve gallic acid (dilutions from 50 to 250 mg GA/L methanol). According to [31], the flavonoid contents were determined with some modifications. Hence, 5 mL of honey solutions (0.01 g of honey/mL distilled water) were combined with the same volume of 2% aluminum chloride solution diluted in methanol and then incubated at room temperature for 30 min. The absorbance was read at 415 nm, and the total flavonoid content was reported as mg of quercetin equivalent (Q.E.) per 100 g of honey [32].

### 2.6. Extraction of Phenolic Compounds for LC-MS-MS Analysis

The extraction process was modified slightly from what was initially reported by Azar et al. [33] and Wahdan [34]. Thus, 10 g of a honey sample was diluted in 50 mL of pure water (20%), and the mixture was stirred for a while to ensure optimum homogenization. The pH of this solution was adjusted to 2 with HCl 0.1 Μ (mol/L). After filtering through absorbent cotton, the phenolic compounds in the honey solution were extracted using 50 mL of ethyl acetate for the first time, followed by 25 mL for the second and third times. At 40 °C, the ethyl acetate extract was evaporated in a rotary evaporator under a vacuum. The residue was placed in 5 mL of methanol and kept at 18 °C. Prior to chromatography, all samples were filtered via Millex-LCR (PTFE) filters with 0.45 m pore sizes.

### 2.7. Liquid Chromatography-Mass Spectrometry Analysis Conditions LC-MS-MS

UPLC-ESI-MS-MS Shimadzu 8040 Ultra-High sensitivity with UFMS technology was employed and equipped with binary bump Nexera XR LC-20AD. For optimization of polyphones standards, we used direct injection without column.

The ESI conditions were as follows: CID gas, 230 KPs; conversion dynode, −6.00 Kv; interface temperature, 350 °C; DL temperature, 250 °C; nebulizing gas flow, 3.00 L/min; heat block, 400 °C; drying gas flow, 15.00 L/min.

All standards were prepared in methanol with a 500 μg/L concentration. The ion trap mass spectrometer was used in both negative and positive ions with MRM mode (multiple reaction monitoring). The mobile phase was constituted of water, 0.1% formic acid, and 70% methanol. The flow rate was 0.3 mL/min, and the injection volume was 6 µL.

The samples were separated using an Ultra-force C18 column (I.D. 2.5 mm ×100 mm, 1.8 µm particle size; Restek), and the oven temperature was 25 °C. Isocratic elution was applied with 0.1% formic acid and methanol (30:70, *v*/*v*). The flow rate was 0.30 mL/min, and the injection volume was 10 mL.

### 2.8. Antioxidant Activity

#### 2.8.1. DPPH Test

The DPPH radical scavenging capacity of the sampled honey was measured following the procedure described by Ferreira et al. [35], with modifications. Thus, 1 ml of honey solution (w/v) was added to 2.7 mL of a methanolic solution containing DPPH radical (0.024 mg/mL). The mixture was stirred with a vortex before being left in the dark for 60 min. The absorbance was then measured at 517 nm against a DPPH-free blank. The data were provided as a percentage of radical DPPH inhibition. The radical inhibition of DPPH was estimated using the following equation: Percentage of Inhibition % = 100 × [(blank absorbance − sample absorbance)/blank absorbance].

#### 2.8.2. FRAP Test

According to Tuberoso et al. [36], the FRAP test was performed with minor modifications.

The ferric reduction antioxidant test (FRAP) uses a spectrophotometric test to lower ferric 2,4,6-tris(2-pyridyl)-1,3,5-triazine [Fe (III)-TPTZ] to the ferric complex at low pH. The FRAP solution is composed of three ingredients: 300 mM sodium acetate, TPTZ (10 mM) diluted in 40 mM HCl, and FeCl_3_ (20 mM). To perform the test, 500 µL of honey solution (0.1 g/1 mL) was combined with 750 µL of FRAP reagent. The absorbance was measured at 593 nm after homogenization and incubation for 5 min at 37 °C. The values are given in milligrams per 100 g of honey.

### 2.9. Antibacterial Activity

#### 2.9.1. Bacterial Strains

The inhibitory effect of the different selected honey samples was tested on five human pathogenic bacterial strains: one is a Gram-negative strain (*Escherichia coli* ATCC 8737), and the others are Gram-positive (*Micrococcus luteus* ATCC 9314, *Bacillus subtilis* ATCC 6633, *Staphylococcus aureus* ATCCN 6538, *Listeria innocua* CLIP 7491). Bacterial strains were kept at −80 °C in brain–heart agar broths containing glycerol until they were used. The strains were grown afterward in agar broths for 24 h at 37 °C in an incubator before being tested for antibacterial activity.

#### 2.9.2. Minimal Inhibitory Concentration (MIC)

The minimum inhibitory concentration (MIC) of honey is defined as the lowest concentration that can prevent bacteria development [37]. The antibacterial activity of the investigated honey samples was examined using the technique performed by Baydar et al. [38]. The disc diffusion method was used to conduct this test, which involves soaking discs in each sample. A pure and young culture (18 h old) should be used to create bacterial suspensions with an optical density of 0.5 Mc Ferland (EQ10^5^UFC/mL). A 1 mL volume of each bacterial strain was inoculated into Petri dishes and then filled with Muller–Hinton agar medium at a thickness of 4 mm, whereby it was then dried for 3 to 5 min at room temperature. Following that, three disks (8 mm in diameter) soaked with the same honey solution concentration were put on the Petri dishes and placed on the growing medium’s surface to achieve full contact with the agar. Next, sterilized control disks were impregnated in a 20 µL volume of distilled water. Petri dishes were then kept for pre diffusion at 4 °C for three hours and then incubated at 37 °C for 24 h. The inhibitory zone diameter was determined in millimeters by using a caliper. For each bacterial strain, the experiment was performed three times.

### 2.10. Statistical Analyses

All tests were carried out in triplicate. Results were reported as mean values with a standard deviation (SD). Microsoft Office Excel 2007 and Pearson’s correlation coefficient (R) using Prism Graph Pad.8 software were used to test correlations between the analytical parameters. ANOVA analyzed data with Tukey test and Matlab vers. 17 for Windows. The level of confidence was set at 95% (α = 0.05). Multivariate PCA analysis was performed using PAST—PAlaeontological STatistics, ver. 1.89 (free software, http://folk.uio.no/ohammer/past, accessed on 14 May 2022).

## 3. Results and Discussion

### 3.1. Vegetation Features

**Figure 2:**
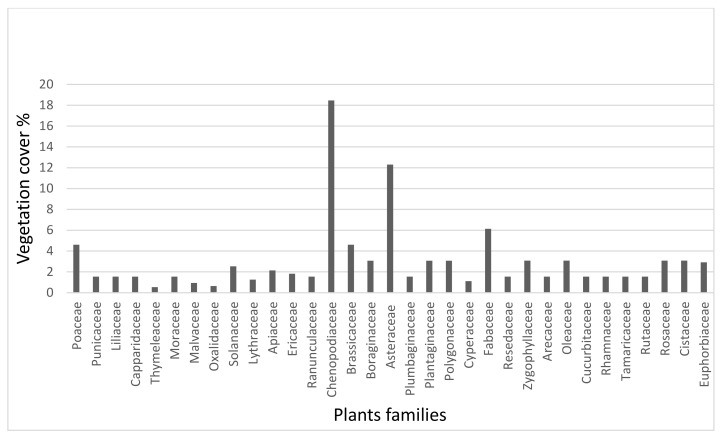
Percentage cover of the prominent botanical families encountered in the study area.

We found 65 species in total, divided into 33 botanical families, where most species belong to Chenopodiaceae (18.46%), followed by Asteraceae (12.307%), Fabaceae (6.135%), Brassicaceae, and Poaceae (4.615%). Boraginaceae, Cucurbitaceae, Plantaginaceae, Polygonaceae, Rosaceae, Solanaceae, and Zygophyllaceae were all under 3.076 percent, with only one species represented (Figure 2). These findings are similar to those published in the exact location [24,39]. The bees visited spontaneous plants, primarily flora, accounting for more than 70% of inventoried plants. Fruit trees, market gardening, fodder crops, and *Phoenix* culture make up the rest.

### 3.2. Qualitative Pollen Analysis

Twenty-three families and thirty-three taxa were identified from the honey samples (Table 2 and Table 3). The taxa Arecaceae, Tamaricaceae, Poaceae, Plantaginaceae, Euphorbiaceae, Ericaceae, Chenopodiaceae, Asteraceae, and *Diplotaxis* have a large distribution (>50%) in the honey samples.

Qualitative pollen analysis highlighted the predominant *Ziziphus lotus* pollen in one honey sample (H02) with a frequency of 48.93%. In general, honey is considered monofloral when the relative pollen frequency of one taxon exceeds 45% [26]. All other kinds of honey are multifloral, showing no pollen as predominant. Moreover, we noted the secondary presence of pollens from Brassicaceae, Asteraceae, and *Citrus*; these taxa constitute an essential source of nectar and pollen.

The pollens of nectarless species such as Arecaceae, Poaceae, Chenopodiaceae, Cistaceae, Plantaginaceae, and Oleaceae were calculated. Although they do not provide nectar, they are essential for pollen in describing the geographical origin [26]. Our results are similar to those from the Steppe region in Algeria, which is also characterized by a hot and dry climate [40,41]. However, Myrtaceae, Apiaceae, and Ericaceae families are most frequently found in honey samples in the Algerian north [42]. Fabaceae, Asteraceae, Apiaceae, and Lamiaceae are the most representative plant groups, accounting for 36% of the total pollen types encountered in honey samples taken from the Kabylia region in Algeria [43].

### 3.3. Physicochemical Properties

Results of the physicochemical properties of the studied honey samples are reported in Table 4.

According to the Tukey test, different letters in the same column indicate highly significant differences (*p* < 0.001) across samples.

Results highlight that the analyzed honey samples are of good quality and clearly within the Codex Alimentarus (2001)-acceptable criteria. Furthermore, these results align with those found in other Algerian honey [42,44,45].

The variance analysis showed a very significant difference (*p* = 0.000, *p* < 0.001) of all the physicochemical analyses tested according to the different types of honey.

The water content varied between 14.200 ± 0.002% to 19.800 ± 0.001%, with an average value of 17.48 ± 1.08%. After sugars, water is the second most prevalent component in honey [46]. The amount of water in the honey is related to several factors: the relative humidity of the harvested season, the level of maturity attained in the hive, processing procedures, and storage conditions. It also varies based on the parent plant’s water content and the nectar and honeydew [47].

The pH value is another crucial factor during honey extraction and preservation. The texture, consistency, and shelf life of honey are all affected by the potential of hydrogen [48].

The honey samples analyzed in this study were acidic (Table 3). pH levels ranged from 3.880 ± 0.015 to 4.373 ± 0.011, with a mean pH value of 4.088 ± 0.127. These values are close to those reported for honey samples from the Kabylia area (Algeria) [43], along with certain Tunisian honey [48], Spanish samples [1], and Malaysian honey [49].

The acidity of honey is due to the presence of organic acid and compounds, such as the content of lactones, esters, phosphates ions, sulfates ions, and chlorides ions [50]. The free-acidity values of all the honey samples ranged between 12.230 ± 0.020 and 27.800 ± 0.050 meq/kg, with an average value of 20.573 ± 3573 meq/kg.

The highest free-acidity values were found in the H05 honey sample (27.800 meq/kg), whereas the lowest free-acidity values were found in the H01 one (12.23 meq/kg). Our findings are consistent with those obtained for various Tunisian honey, which ranged from 7.11 to 27.70 meq/kg [48], whereas Otmani et al. [51] found that the free acidity of two honey samples collected in northern Algeria was higher (37 and 41 meq/kg). The acidity variations have been attributed to flowers’ origin and the harvesting season [52].

Electrical conductivity (E.C.) strongly correlates with organic acids, proteins, mineral or total ash concentrations, and salts. It is a characteristic that varies greatly depending on the honey’s floral origin [48,51]. Electrical conductivity values should be less than 0.8 mS cm^−1^ for floral honey, whereas values for honeydew should be greater than 0.8 mS/cm [53]. All of the results (except for the H09 sample, in which the E.C. value was greater than 0.8 mS/cm) were below the required maximum level of electrical conductivity for honey (<0.800 mS/cm).

Honey comes in various colors, ranging from pale yellow to amber and dark amber and from dark amber to black, with some rare shades of green and red in severe situations or even the color red [9].

According to Pfund’s index, the color values obtained vary from 47.462 ± 0.0302 to 171.135 ± 0.003 mm, with an average value of 106.216 ± 38.847 mm (Table 4); 10% of the honey samples examined, including only one sample, were extra-light amber in color, and 20% were a dark color. Moreover, 30% of samples were dark amber, and 40% (four samples) were light amber. Our results of Pfund’s index are similar to those of Ghorab et al. [43] (37 to 135 mm) and Frankel et al. [54] (31.12 to 166.68 mm).

Several authors have demonstrated that the number of phenols, minerals, and acids is much higher in dark honey than in light honey; thus, a robust antioxidant activity has been registered in dark honey [49,54].

### 3.4. Total Polyphenols and Flavonoids Results

Results of the polyphenol contents, flavonoids, and antioxidant capacities of the tested sample honey are presented in Table 5.

The variance analysis showed a very significant difference (*p* = 0.000, *p* < 0.001) of all the antioxidants tested according to the different types of honey.

According to the Tukey test, different letters in the same column indicate highly significant differences (*p* < 0.001) across samples.

#### Total Phenolic Content

Polyphenols are essential components of honey found in tiny amounts and generated from the pollen of plants frequently visited by bees [55]. The total phenolic compounds in our honey samples varied from 44.186 ± 0.006 to 508.536 ± 0.006 mg/kg, whereas the average total phenolic level ranging from 41.800 mg GAE/100 g to 128.300 mg GAE/100 g observed in honey [56] was less than those obtained in our findings. Moreover, these samples contained more than those found in the Malaysian regions [57], with reported phenolic content values of honey samples in an interval between 110.394 mg GAE/100 g and 196.500 mg GAE/100 g. On the other hand, Dżugan et al. [56] found that the total phenolics in Polish honey varied from 205.41 to 1353.66 mg GAE/100 g.

Plant secretions are the primary source of phenolic chemicals, which are secondary metabolites; the difference in total phenolic content might be attributable to the geographical location of the various floral sources [58,59].

Flavonoids are phenolic chemicals with a low molecular weight that give honey its fragrance and antioxidant properties [49]. The total flavonoid content was found to be 105.088 ± 68.808 mg Q.E./100 g per sample of honey on average, with a low value of 20.444 ± 0.012 mg Q.E./100 g per sample of honey and a high value of 338.558 ± 0.002 mg Q.E./100 g per sample of honey (Table 5). These honey specimens have a more excellent content of flavonoids than Burkinafassou honey [58], Italian honey [59], and Algerian honey tested by Khalil et al. [60].

### 3.5. Phenolic Compounds LC-MS-MS Analysis

The chromatography results showed the presence of 20 different phenolic compounds across the board for all honey samples (Table 6).

Myricetin, verbascoside, esculin, rutin, and chrysin were found in all honey samples. However, acetylsalicylic acid, *p*-coumaric acid, and gallic acid were detected only in honeydew honey (H09) and which is the richest in phenolic compounds. These results align with those obtained by Trautvetter et al. [61].

### 3.6. Antioxidant Activity

#### 3.6.1. DPPH Test

DPPH radical scavenging activity showed a significant variance (Table 5). Antioxidant levels of the tested honey samples ranged from 3.117 ± 0.090 to 23.950 ± 0.050 mg/mL. The sample H08 honey had considerably greater antiradical ability (3.117 mg/mL) than the other honey samples. The lowest capacity was recorded in the sample H02 (23.950 mg/mL). The lowest IC_50_ value implies a remarkable ability to scavenge free radicals. Meda et al. [58] found a similar antioxidant activity that ranged from 1.630 to 29.130 mg/mL, which was lower than monofloral Turkish honey samples, with values ranging from 12.010 to 65.520 mg/mL [62].

The difference between the antioxidant capacity values of the tested honey samples may be due to their antioxidants’ nature and the quality and quantity of their phenol content [17].

#### 3.6.2. FRAP Test

The FRAP test is based on the capacity to decrease ferrous iron Fe^3+^ to ferric iron Fe^2+^ by using antioxidants. Power reduction is one of the antioxidant processes [63]. According to the results recorded in Table 5, the values of antioxidant activity by the FRAP test of the honey samples vary from 44.186 ± 0.030 to 570.731 ± 0.070 (μM Fe(II)/Kg). This interval is similar to that obtained by Gül et Pehlivan [62].

#### 3.6.3. Correlation Analysis

Correlation analysis between the studied honey sample parameters underlined highly significant differences. A higher negative correlation between phenolic content and DPPH (r = −0.9504, *p* < 0.0001), phenolic content and FRAP (r = −0.9568, *p* < 0.0001), and between phenolic content and color (r = −0.9666, *p* < 0.0001) were discovered. Our correlation coefficients are in perfect accordance with those obtained in the study of Boussaid et al. [48]. A negative correlation also was observed between DPPH and color (r = 0.8917, *p* = 0.0005), and these findings are similar to those mentioned by Baltrušaitytė et al. [64] (r = −0.716). The positive correlation between FRAP and DPPH values was statistically significant (r = 0.9648; *p*< 0.0001). However, compared to Perna et al. [59] (r = 0.61), this correlation coefficient was significantly greater.

#### 3.6.4. Antibacterial Results

New treatment techniques are required due to pathogenic bacteria’s increasing antibiotic resistance and a shortage of therapeutic choices [65,66].

Honey’s natural components have a variety of antimicrobial properties against various bacteria. Honey’s antibacterial action is believed to be affected by the pasture where the bees were reared, climatic circumstances, and blossom nectar’s natural composition [19,66].

**Figure 3:**
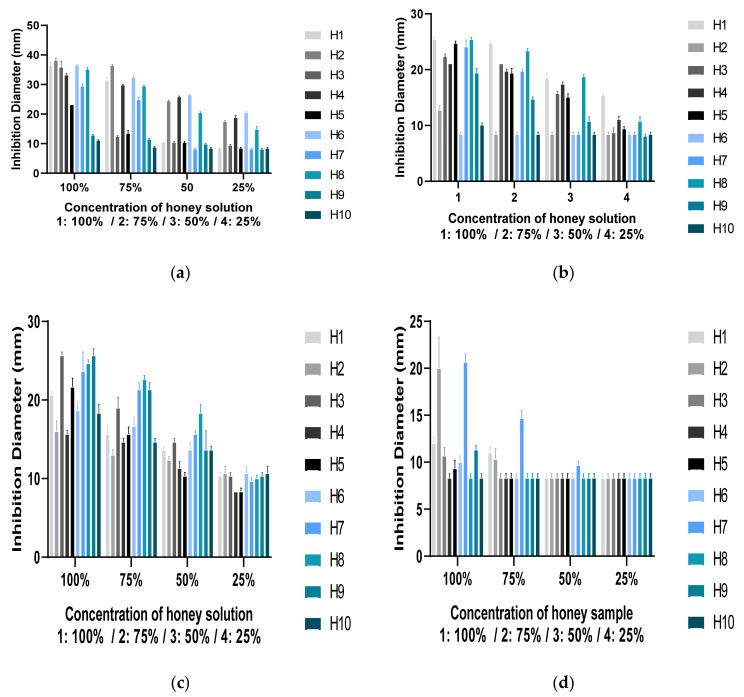

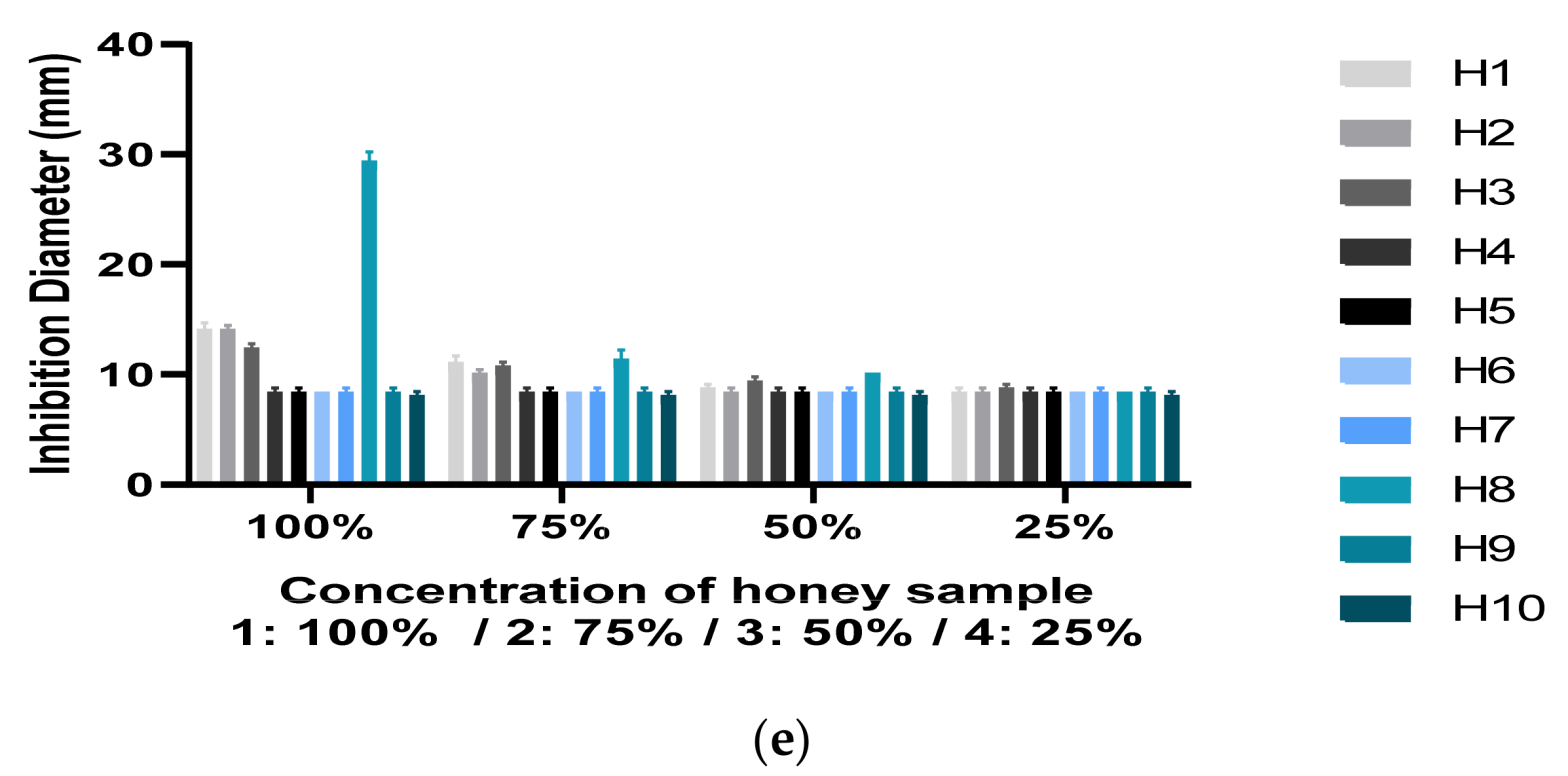
Antibacterial activity of honey samples against: (**a**) *S. aureus* (S2); (**b**) *E. coli* (S1); (**c**) *L. innocua* (S3); (**d**) *B. subtilis* (S4); (**e**) *M. luteus* (S5).

Figure 3 and Table 7 compare the antibacterial activity of different honey samples at four concentrations (100%, 75%, 50%, and 25% *w/v*) against Gram-positive and Gram-negative bacteria by well diffusion experiments.

Generally, all studied kinds of honey were efficient against *Staphylococcus aureus* and *Listeria innocua.* The H01, H02, and H03 honey samples inhibited all pathogens examined. Staphylococcus aureus was the most affected Gram-positive bacteria with the most considerable inhibitory zone impact.

*Bacillus subtilis* and *Micrococcus luteus* were not inhibited by H04 and H10 honey samples. Except for H06, all concentrations of the tested honey samples inhibited *E. coli*. Honey exhibited a more potent antibacterial effect on three Gram-positive pathogens than on Gram-negative bacteria. (Table 7). The H02 sample had the best zone of inhibition against *S. aureus*, with an inhibition diameter measured from 38.66 ± 0.88 to 17.33 ± 0.44 mm (100% to 25%) (Figure 3a). The best effect on the *E. coli* strain was observed in the H 01 sample, with an average diameter ranging from 25.33 ± 0.44 to 15.33 ± 0.44 mm (Figure 3b). *Listeria innocua* was more sensitive to H03 than the other honey samples, with a sensitivity interval of 25.66 ± 0.44 to 10.33 ± 0.44 mm (Figure 3c). *Bacillus subtilis* and *Micrococcus luteus* were the most resistant to the other pathogenic bacteria, whereby the best inhibition for *B. subtilis* was noticed in samples H02 and H07 (20.66 ± 0.88 to 10.33 ± 1.11 mm) (Figure 3d). For *M. luteus,* a diameter of 29.33 ± 0.88 to 10 ± 0.00 mm was obtained with the H08 sample effect (Figure 3e).

Table 6 presents the MIC values of the studied honey samples against all tested pathogenic bacterial strains. MIC values range from 5% to 100%. According to the minimum inhibitory concentration (MIC) findings, *S. aureus* was the most sensitive bacteria, while *M. luteus* and *B. subtilis* were resistant. (Table 7). Honey was also found to be efficient against Gram-positive (*S. aureus, Bacillus subtilis, Bacillus cereus, Enterococcus faecalis*, and *Micrococcus luteus*) and Gram-negative (*Escherichia coli*, *P. aeruginosa*, and *Salmonella typhi*) bacteria [67]. Additionally, according to many studies, *S. aureus* has a high susceptibility [68,69] compared to Gram-negative bacteria. In contrast, the Ukrainian honey samples were efficient against *Listeria monocytogenes* ATCC 7644 and could not inhibit *Staphylococcus aureus* CCM 4223 growth [18,70].

The variance analysis showed a very significant difference in the variation of inhibition diameter according to the different honey types, the strains and concentrations, and the interaction between factors, with probability values (*p* = 0.000; *p* < 0.001).

**Figure 4:**
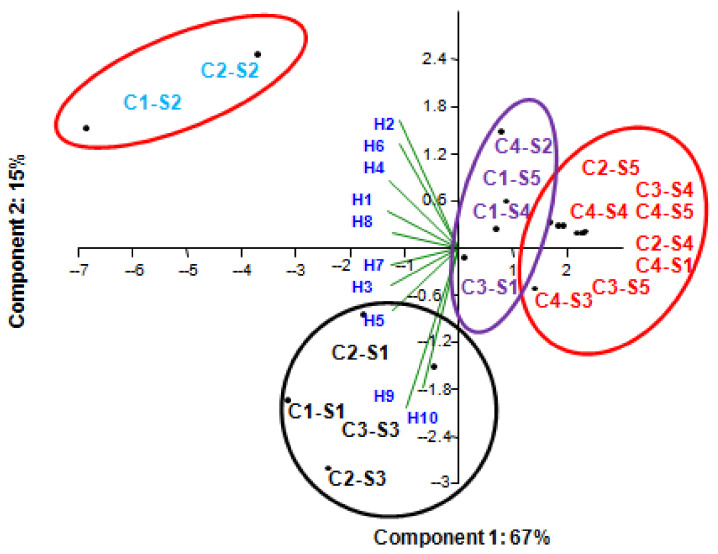
A plot of the two first components of the PCA of antibacterial activity. C1: 100%, C2: 75%, C3: 50%, C4: 25%, S1: *E. coli*, S2: *S. aureus*, S3: *L. innocua*, S4: *B. subtilis*, S5: *M. luteus*.

The principal component analysis (PCA) is satisfactory for the studied parameters (honey samples, bacterial strains, and concentration factor) since more than 80% of the variance is expressed on the first two axes (Figure 4). The PCA vertical axis vertical explained 67% of the total variance while the horizontal axis, a further 15% as well as the APC highlighted four groups: the first one, Group A, was constituted by the C1_2, and C2_S2 is correlated with the following honey samples: H02, H06, H04, H01, and H08.

The second group correlated C1_S1, C2_S1, C2_S2, and C3_S3 with H03, H05, H09, and H10. At the same time, no correlation was recorded in the other two groups with the honey samples.

## 4. Conclusions

High-quality honey production is feasible given the floristic biodiversity in Algeria. This research has led to the identification of an extensive range of melliferous plants in the pre-Saharan zone, with most of them are spontaneous species. Overall, the results of the physicochemical analysis revealed that the Algerian honey samples from this region were of outstanding quality, conforming to international standards and having a composition mainly determined by their botanical origin. Furthermore, all honey samples demonstrated potent antioxidant and antibacterial capabilities. This could be due to the high polyphenol and flavonoid content. As a result, we propose that these plants should be preserved and protected, mainly because some of them are beneficial to human health and the ecosystem.

## Figures and Tables

**Table 1:** Geographical Origin of the honey samples.

Samples’ Number	Study Sites	Longitude	Latitude	Altitude
H01	Ben Guecha	7°18′28.1″ E	34°12′07.2″ N	28 m
H02	Oued El Alenda	6°45′43.6″ E	33°14′01.1″ N	105 m
H03	Guemar	6°49′47.1″ E	33°32′06.7″ N	61 m
H04	El Megrane	6°51′49.6″ E	34°04′56.0″ N	70 m
H05	Debila	6°57′16.5″ E	33°31′22.8″ N	65 m
H06	Reguiba	6°42′44.4″ E	33°33′55.6″ N	59 m
H07	Ourmas	6°46′38.4″ E	33°24′22.5″ N	80 m
H08	Hassi Khalifa	7°01′31.8″ E	33°42′24.6″ N	31 m
H09	El Meghaier	5°54′33.4″ E	33°58′23.6″ N	02 m
H10	Ogla	6°59′42.7″ E	33°08′50.1″ N	89 m

**Table 2:** Frequency classes of pollen types in the studied honey samples.

Family	Pollen Type	%	Pollen Class				Max %
			Predominant (P)	Secondary (S)	Important Minor (I)	Minor (M)	
*Apiaceae*	*Thapsia*	20	---	---	---	2	1.98
Arecaceae *	Phoenix dactylifera *	90	---	---	5	2	5.23
*Asteraceae*	*Echinops*	50	---	---	---	3	1.33
	*Centaurea*	30	---	---	3	2	8.11
	*Scolymus*	40	---	---	1	2	3.65
	*Calendula*	30	---	---	2	3	10.58
	*Asteraceae*	80	---	3	5	---	30.74
*Boraginaceae*	*Echium*	10	---	---	---	1	1.95
	Boraginaceae	30	---	---	1	2	6.94
*Brassicaceae*	*Diplotaxis*	60	---	---	3	---	15.42
	Brassicaceae	30	---	2	2	1	33.51
Chenopodiaceae *	Chenopodiaceae *	70	---	---	3	3	3.80
Cistaceae *	Cistus *	30	---	---	2	1	4.35
*Ericaceae*	Ericaceae	60	---	---	1	5	13.39
*Euphorbiaceae*	*Euphorbia* sp.	60	---	---	5	3	15.5
*Fabaceae*	*Ononis*	10	---	---	---	1	1.39
	*Retama retam*	50	---	---	5	---	16.54
	*Acacia*	10	---	---	---	1	1.23
	Fabaceae	50	---	---	5	---	16.54
*Liliaceae*	Liliaceae	10	---	---	1	---	8.76
*Malvaceae*	*Malva*	10	---	---	1	---	3.12
*Oleaceae*	Olea europea *	80	---	---	---	8	2.88
Plantaginaceae *	Plantago *	80	---	---	5	3	5.95
Poaceae *	Poaceae *	90	---	---	3	6	7.60
*Oxalidaceae*	*Oxalis*	10	---	---	---	1	2.53
*Resedaceae*	*Reseda alba*	40	---	---	1	3	5.23
*Ranunculaceae*	Ranunculaceae	20	---	---	---	2	1.20
*Rhamnaceae*	*Ziziphus lotus*	30	1	---	2	---	48.93
*Rosaceae*	Rosaceae	50	---	---	1	4	6.24
*Rutaceae*	*Citrus*	20	---	2	---	---	20.9
*Tamaricaceae*	*Tamarix*	90	---	---	2	6	8.97
*Zygophylaceae*	*Peganum harmala*	30	---	---	2	1	3.65

*, nectarless species; Max, maximum recorded pollen frequency; %, percentage of the presence of each pollen type in honey samples; P, >45%; S, 16–45%; I, 3–15%; M, 1–3%.

**Table 3:** Qualitative pollen analysis of honey samples.

Honey Samples	Pollen Type Classes			
	>45%	16–45%	3–16%	<3%
H01		*Brassicaceae*	*Scolymus*, *Calendula*, Chenopodiaceae, *Plantago*, *Phoenix dactylifera*, *Retama retam*, Boraginaceae	Ericaceae, *Reseda alba*, Poaceae, *Tamarix*, *Olea europea*, *Echinops*
H02	*Ziziphus lotus*		Ericaceae, *Cistus*, Asteraceae, *Plantago, Phoenix dactylifera*	Boraginaceae, Chenopodiaceae, *Peganum harmala*, *Tamarix*, *Poaceae*, *Thapsia*, *Euphorbia* sp., *Brassicaceae*, *Calendula*, *Echinops*, *Olea europea*, *Phoenix dactylifera*, Poaceae, Rosaceae, Rhenonculaceae
H03		*Asteraceae*	*Centaurea,* Fabaceae, Poaceae, *Plantago*, *Malva*, *Euphorbia* sp., Asteracreae, Chenopodiaceae	*Phoenix dactylifera*, *Oxalis*, Brassicaceae, *Euphorbia* sp., *Olea europea*, *Tamarix*
H04			*Retama retam*, *Cistus*, *Peganum harmala*, *Euphorbia* sp., *Diplotaxix*, Asteraceae, *Ziziphus lotus*, *Plantago*, Poaceae, *Phoenix dactylifera*	Chenopodiaceae, Ericaceae, *Tamarix, Thapsia, Echinops, Olea europea,* Ranunculaceae, Rosaceae, *Calendula*
H05		Brassicaceae, *Citrus*	*Ziziphus lotus*, *Calendula*, *Phoenix dactylifera*, *Euphorbia* sp., *Peganum harmala*, *Tamarix*	*Thapsia*, *Ononis*, *Echinops*, *Echium*, *Cistus*, Ericaceae, *Plantago*, *Olea europea*, *Rosaceae*
H06		*Asteraceae*	*Calandula*, Asteraceae, Rosaceae, *Euphorbia* sp., *Plantago*, Boraginaceae, *Diplotaxix*	*Cistus*, Renonculaceae, *Thapsia*, *Echinops*, Poaceae, Rosaceae, *Centaurea*, Brassicaceae
H07		*Citrus*	Asteraceae, Brassicaceae, *Euphorbia* sp., Fabaceae, *Retama retam*, *Phoenix dactylifera*	Chenopodiaceae, Poaceae, *Reseda alba*, *Tamarix*, *Olea europea*, *Scolymus*, *Poaceae*, *Calendula*
H08		*Asteraceae*	*Centaurae*, Brassicaceae, *Plantago*, *other fabaceae*, *Liliaceae*, *Diplotaxix*	*Boraginaceae*, *Olea europea*, *Tamarix*, *Reseda alba*, *Euphorbia* sp., *Phoenix dactylifera*, Ericaceae, Poaceae, Rosaceae
H09		*Asteraceae*	Brassicaceae, Fabaceae, Poaceae, *Reseda alba*, Rosaceae, *Tamarix*, *Reatama retam*	*Centaurea*, Ericaceae, *Plantago*, *Olea europea*, *Thapsia*, *Acacia*, *Calendula*
H10			Asteraceae, *Centaurea*, *Retama retam*, Fabaceae, *citrus*, *Poaceae*, *Phoenix dactylifera*, Chenopodiaceae	*Scolymus*, *Euphorbia* sp., *Plantago*, *Tamarix*, *Olea europea*

**Table 4:** Physicochemical characteristics of the studied honey samples (different superscripts letters indicate a statistically significant difference between values).

Honey Samples	pH	Free Acidity (meq/kg)	Water Content (%)	Electrical Conductivity (mS/cm)	Color (Pfund Index)
H01	4.102 ± 0.013 ^d^	12.230 ± 0.020 ^b,c^	17.800 ± 0 ^b,c,d^	0.213 ± 0.005 ^g^	47.462 ± 0.030 ^i^
H02	3.944 ± 0.008 ^g^	25.300 ± 0.050 ^b,c^	18.600 ± 0.001 ^e^	0.254 ± 0.001 ^f,g^	109.856 ± 0.002 ^g^
H03	4.203 ± 0.011 ^c^	15.500 ± 0.090 ^a^	16.600 ± 0001 ^b^	0.235 ± 0.020 ^a^	159.993 ± 0.005 ^b^
H04	4.006 ± 0.017 ^e^	21.200 ± 0.060 ^g^	17.400 ± 0.006 ^bc^	0.240 ± 0.002 ^e,f,g^	80.144 ± 0.001 ^f^
H05	3.991 ± 0.013 ^e^	27.800 ± 0.050 ^b^	18.600 ± 0.001 ^c,d,e^	0.275 ± 0.004 ^d,e^	70.860 ± 0.002 ^g^
H06	4.016 ± 0.017 ^e^	20.820 ± 0.050 ^c^	19 ± 0.004 ^d,e^	0.268 ± 0.007 ^d,f,e^	129.911 ± 0.001 ^d^
H07	4.313 ± 0.011 ^a^	18.600 ± 0.020 f	18.600 ± 0.006 ^a^	0.562 ± 0.008 ^d^	154.422 ± 0.003 ^c^
H08	4.373 ± 0.011 ^b^	16.980 ± 0.020 ^e^	14.200 ± 0.002 ^c,d,e^	0.290 ± 0.019 ^b^	171.135 ± 0.003 ^a^
H09	3.884 ± 0.015 ^f^	27.300 ± 0.080 ^e,f^	19.800 ± 0.001 ^c,d,e^	0.948 ± 0.005 ^d,e,f^	70.488 ± 0.006 ^e^
H10	4.086 ± 0.004 ^d^	20.000 ± 0.080 ^d^	17.800 ± 0.002 ^b,c,d^	0.520 ± 0.012 ^c^	67.888 ± 0.002 ^h^
Mean ± SD	4.088 ± 0.127	20.573 ± 3,57	17.48 ± 1.08	0.381 ± 0.177	106.216 ± 38.84
*F*-value	234.72	253.60	20.69	859.22	9458.41

**Table 5:** Phenolic content (mg GAE/100 g), total flavonoid (mg Q.E./100 g), FRAP (μM Fe (II)/Kg), and DPPH (mg/mL) IC_50_ values of the studied honey samples (different superscripts letters indicate a statistically significant difference between values).

Honey Samples	Total Phenolic Content (mg GAE/100 g)	Total Flavonoids (mg QE/100 g)	DPPH, IC_50_ (mg/mL)	FRAP Assay (μM Fe(II)/Kg)
H01	44.186 ± 0.006 ^j^	36.111 ± 0.004 ^h^	10.390 ± 0.040 ^a^	44.186 ± 0.030 ^a^
H02	75.609 ± 0.006 ^h^	48.777 ± 0.177 ^f^	23.950 ± 0.050 ^c^	75.609 ± 0.080 ^c^
H03	508.536 ± 0.006 ^b^	215.606 ± 0.128 ^b^	4.070 ± 0.080 ^j^	508.536 ± 0.070 ^h^
H04	289.635 ± 0.005 ^f^	20.444 ± 0.012 ^i^	10.490 ± 0.060 ^e^	289.634 ± 0.060 ^e^
H05	215.630 ± 0.001 ^g^	101.666 ± 0.012 ^c^	8.140 ± 0.080 ^d^	215.630 ± 0.070 ^d^
H06	318.097 ± 0.007 ^d^	74.888 ± 0.132 ^e^	6.395 ± 0.030 ^f^	381.097 ± 0.040 ^f^
H07	507.731 ± 0.006 ^c^	338.558 ± 0.002 ^d^	5.504 ± 0.030 ^g^	459.552 ± 0.030 ^g^
H08	459.552 ± 0.001 ^a^	94.777 ± 0.004 ^a^	3.117 ± 0.090 ^i^	570.731 ± 0.070 ^i^
H09	353.252 ± 0.001 ^e^	74.444 ± 0.012 ^e^	11.901 ± 0.070 ^h^	353.252 ± 0.030 ^e^
H10	63.617 ± 0.001 ^i^	45.555 ± 0.003 ^g^	6.677 ± 0.060 ^b^	63.617 ± 0.020 ^h^
Mean ± SD	296.184 ± 158.440	105.088 ± 68.800	9.063 ± 4.090	123.796 ± 45.290
*F*-value	163,983.77	28,334.9	39,962.48	10,479.04

**Table 6:** LC-MS-MS-determined phenolic compounds of honey samples.

Compound Name.	Charge +/−	Precursor *m*/*z*	Product *m*/*z*	H01	H02	H03	H04	H05	H06	H07	H08	H09	H10
Acetylsalicylic Acid	[MH]^+^	181.1	98.59 131	ND	ND	ND	ND	ND	ND	ND	ND	D	ND
Cinnamic Acid	[MH]^+^	149.1	77.2	D	D	D	D	ND	D	ND	D	D	D
*p*-Coumaric Acid	[MH]^+^	165.1	59.1	ND	ND	ND	ND	ND	ND	ND	ND	D	ND
Gallic Acid	[MH]^−^	168.8	125.1	ND	ND	ND	ND	ND	ND	ND	ND	D	ND
Caffeic Acid	[MH]^−^	178.8	135.1	D	D	D	D	D	D	D	ND	D	D
Chlorogenic Acid	[MH]^+^	355	73.15	D	D	D	ND	D	D	D	D	D	D
Chrysin	[MH]^+^	255.1	223.3 207.25	D	D	D	D	D	D	D	D	D	D
4-Hydroxycoumarin	[MH]^−^	160.8	117.1	D	D	D	ND	D	D	ND	ND	ND	D
Esculin	[MH]^+^	341.3	309.4	D	D	D	D	D	D	D	D	D	D
Butylhydroxyanisole	[MH]^+^	181.1	99.15 81.05	ND	D	D	D	D	ND	D	D	D	ND
Kaempferol	[MH]^+^	287.1	255.25	D	D	D	D	D	D	ND	D	D	D
Lawsone	[MH]^+^	175.1	134.2	D	D	D	ND	D	D	D	D	D	D
Naringenin	[MH]^+^	273.1	191.1 232.2	D	D	D	D	D	D	D	D	D	ND
Quercetin	[MH]^+^	303.1	262.2	ND	D	D	D	D	D	D	D	D	D
Resorcinol	[MH]^+^	111.1	79.15	D	D	ND	D	D	ND	D	D	D	D
Rutin	[MH]^+^	611.2	73.2	D	D	D	D	D	D	D	D	D	D
Vanillin	[MH]^+^	153.1	71.15	ND	ND	ND	D	D	ND	D	D	D	D
Verbascoside	[MH]^+^	625.2	593.4	D	D	D	D	D	D	D	D	D	D
Butylated hydroxytoluene	[MH]^+^	221	161.3 203.25	ND	ND	D	ND	ND	D	D	D	D	ND
Myricetin	[MNH_4_]^+^	336.2	46.15	D	D	D	D	D	D	D	D	D	D

D, detected; ND, not detected.

**Table 7:** Inhibition % of different honey samples against bacterial strains.

Honey Sample/Bacteria Strain	*E. coli*	*S. aureus*	*L. innocua*	*B. subtilis*	*M. luteus*
H01	5%	50%	25%	75%	75%
H02	75%	5%	25%	75%	75%
H03	2%	25%	25%	100%	75%
H04	25%	5%	50%	N I	N I
H05	25%	50%	50%	100%	N I
H06	N I	5%	25%	100%	N I
H07	75%	50%	25%	50%	N I
H08	25%	5%	25%	N I	50%
H09	50%	5%	25%	100%	N I
H10	100%	75%	25%	N I	N I

N I, not inhibited. Zone Inhibition ≥ 10 mm.

## Data Availability

Data is available.
